# Supplementary material for: Targeting a cell surface vitamin D receptor on tumor-associated macrophages in triple-negative breast cancer
Source: eLife. 2021 Jun 1;10:e65145. doi: 10.7554/eLife.65145 (PMC8169110; doi:10.7554/eLife.65145)
Supplement: Supplementary file 2. [file elife-65145-supp2.docx]

| **Parameter** | **Values** | **Units** |
| --- | --- | --- |
| $\sigma$ | $0.552$ | day^-1^ |
| $\lambda$ | $6.70\cdot{10}^{4}$ | mm^3^day^-1^(mg/mm^3^)^-1^ |
| $D_{\mathrm{Pep}}$ | $0.985$ | mm^3^day^-1^ |
| $K_{d}$ | $18.7$ | mM |
| $k_{\mathrm{ex}}^{\mathrm{Pep}}$ | $3.31\cdot{10}^{-4}$ | day^-1^ |
| $V_{0}$ | $1.56\cdot{10}^{-2}$ | mm^3^ |
| $k_{a}^{\mathrm{GCV}}$ | $7.44$ | day^-1^ |
| $k_{\mathrm{ex}}^{\mathrm{GCV}}$ | $535.2$ | day^-1^ |
